# National Institutes of Health Blueprint Neurotherapeutics Network: Results to Date and Path Forward

**DOI:** 10.1007/s13311-017-0530-2

**Published:** 2017-04-12

**Authors:** Charles L. Cywin, Amir P. Tamiz

**Affiliations:** Division of Translational Research, National Institute of Neurological Disorders and Stroke, National Institutes of Health, Bethesda, MD USA

Neuroscience encompasses a wide variety of diseases and spans the missions of many of the National Institutes of Health (NIH) Institutes and Centers (ICs). To address the need for crosscutting neuroscience research, the NIH established the NIH Blueprint for Neurological Research (http://neuroscienceblueprint.nih.gov/) comprised of 15 separate NIH ICs whose missions have neuroscience components. With many biopharmaceutical companies exiting or significantly downsizing their neuroscience efforts in the last decade, it highlighted the need for NIH to provide a catalyst for neuroscience drug discovery efforts [1, 2]. To foster small-molecule neurotherapeutic development, the NIH Blueprint Neurotherapeutics Network (BPN) (http://neuroscienceblueprint.nih.gov/bpdrugs/) was launched in 2011, creating a first-of-its-kind virtual pharma network at NIH (Fig. 1) [3]. The BPN is a milestone-driven cooperative agreement program bringing together a unique blend of grant dollars, industry-standard scientific expertise, and contract resources to foster drug development from the discoveries of academic and small businesses with the goal of advancing at least one compound into clinical trials by the end of the first 5 years.

**Fig. 1:**
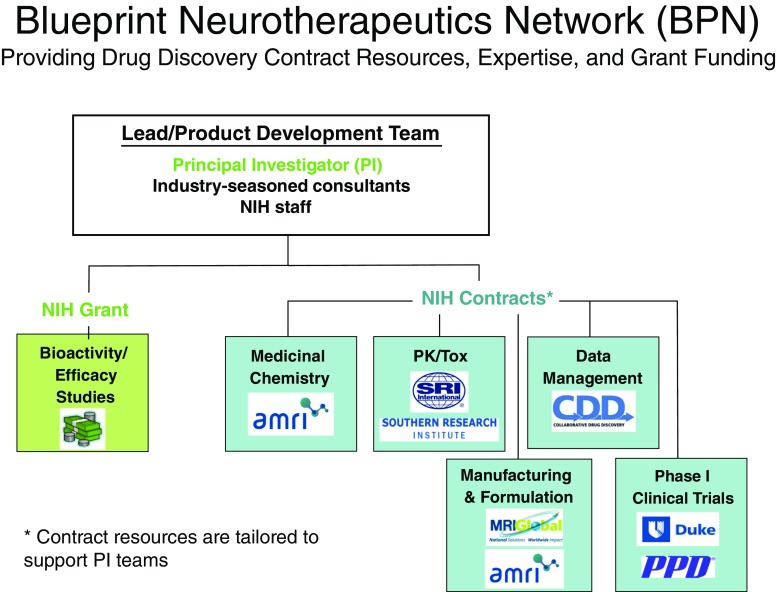
Network resources. NIH = National Institutes of Health; PK/Tox = pharmacokinetics/toxicology

The BPN’s initial target was to start up to 20 milestone-driven projects and advance those projects with the most promise of obtaining Food and Drug Administration approval to enter and execute phase I clinical trials with BPN resources. Another explicit goal of the BPN was to de-risk the development of new, impactful therapies that could attract commercial interest enabling a hand-off to industry for late-stage development and additional clinical trials. Therefore, careful consideration was given in the program to ensure that intellectual property of the contributing academic institution or small business was protected and unencumbered by the NIH in order to simplify licensing discussions with pharma and venture capital investors.

As a first-of-its-kind program at NIH, we projected prelaunch a 50% attrition at each stage considering an overall lower success rate for central nervous system drugs, limited NIH resources, and the average industry attrition rates [4]. In practice, each application round was reviewed by a National Institute of Neurological Disorders and Stroke Special Emphasis Panel consisting of members with disease expertise, as well as drug discovery and development experts with industry experience in multiple disciplines. The BPN launched 15 projects over the period 2011 to 2013, directed at 13 different disease indications in the mission of 7 different BPN ICs (listed in Table 1). The BPN formed customized Lead Discovery Teams around each of the grantees’ project needs and worked in a collaborative fashion, typically meeting every 2 weeks via web-enabled meetings. Through these meetings, progress was measured against milestones, and scientific direction was altered collaboratively based on emerging data in order to give the teams the best chance at meeting their milestones and advancing further. Formal milestone reviews with go/no go decisions for continued funding typically occurred once or twice a year depending on the stage of the program. Additionally, as projects progressed, Lead Discovery Teams met face-to-face at major transition points such as start of Lead Optimization, Start of Development and Investigational New Drug preparation. The regular interactions allowed for maximum benefit of the BPN infrastructure and consultants’ expertise to the grantees, as well as focusing efforts on critical path activities. It should be noted that projects from academic institutions were highly represented throughout the stages of the program (60% of projects at the onset, 80% of projects reaching the preclinical safety stage) demonstrating the quality of the starting projects and success of our mentoring from our NIH staff and consultants.

**Table 1:** Portfolio of Blueprint Neurotherapeutics Network projects starting 2011–13

Institution	Grant title	NIH IC
Columbia University Health Sciences	Small-molecule drugs for treatment of dry age-related macular degeneration	NEI
Navigen, Inc.	Targeting cytokine-mediated pathologies for neuroprotection in treatment of AMD	NEI
University of Miami School of Medicine	Triazine-based compounds to promote regeneration in optic neuropathies	NEI
Axerion Therapeutics, Inc.	Small-molecule development of PrPc antagonists for the treatment of Alzheimer’s disease	NIA
Tetra Discovery Partners, Inc.	PDE4D allosteric modulators for treating cognitive impairment	NIA
University of California San Diego	Optimization of soluble gamma-secretase modulators for the treatment of Alzheimer’s disease	NIa
Sage Therapeutics, Inc.	Neuroactive steroid GABA_A_ receptor positive modulators for fragile X syndrome	NICHD/NIMH/NINDS
Scripps Florida/Eolas Therapeutics, Inc.	Orexin receptor antagonists for drug addiction and panic disorder	NIDA
University of Washington	Drug discovery for the prevention of hearing loss	NIDCD
Trevena, Inc.	A novel delta opioid receptor-biased agonist for major depressive disorder	NIMH
Brigham and Women’s Hospital	Small-molecule modulators of the glutamate transporter for treatment of ALS	NINDS
Emory University	EP2 allosteric Potentiators for subarachnoid hemorrhage	NINDS
Massachusetts General Hospital	Optimization of compounds to improve mRNA splicing in familial dysautonomia	NINDS
Northwestern University at Chicago	A novel calcium channel antagonist for neuroprotection in Parkinson’s disease	NINDS
Reset Therapeutics, Inc.	Orexin receptor agonists for the treatment of excessive daytime sleepiness and cataplexy	NINDS

NIH IC = National Institutes of Health Institutes11111 and Centers; NEI = National Eye Institute; AMD = age-related macular degeneration; NIA = National Institute on Aging; GABA = γ-aminobutyric acid; NICHD = National Institute of Child Health and Human Development; NIMH = National Institute of Mental Health; NINDS = National Institute of Neurological Disorders and Stroke; NIDA = National Institute on Drug Abuse; NIDCD = National Institute on Deafness and Other Communication Disorders; ALS = amyotrophic lateral sclerosis

The BPN worked closely with the teams to establish the viability of the chemical matter and the assays to drive structure–activity–relationship (SAR) studies. Six projects failed to meet their initial set of milestones in a timely fashion for advancement and further SAR studies (Fig. 2). The majority of the failures resulted from the lack of reproducible activity upon re-synthesis or a lack of exploitable SAR of the chemical matter. Of the 9 projects that underwent the chemistry optimization phase, 2 did not achieve the first-year optimization milestones, 1 project withdrew from the program owing to changing business priorities during the Hit-to Lead phase, and an additional project failed to achieve its milestones fully within the time constraints of the BPN in the Lead Optimization phase. Although these projects still had promise, the limited resources and strategic focus of the BPN program did not allow going back to the starting point of the project to re-evaluate additional chemical matter or to develop completely new assays.

**Fig. 2:**
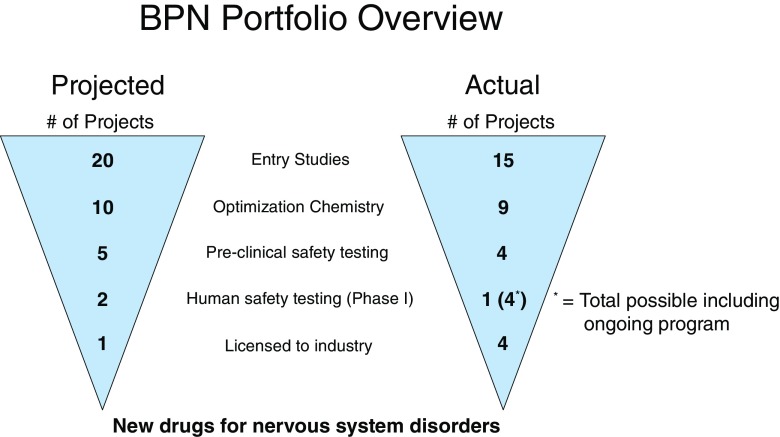
Progression of projects. BPN = Blueprint Neuortherapeutics Network

However, the BPN did encourage the principal investigators (Table 1) to continue promising avenues and, in the case of the hearing-loss program at the University of Washington, it continued with non-BPN NIH funding, and the team was able build on the BPN results to address the underlying issues, garner outside funding, and launch a new company for further development [5]. A second project from Reset Therapeutics targeting excessive daytime sleepiness and cataplexy recently established a research collaboration with Alkermes [6]. The reasons for not reaching the milestones in the Hit-to-Lead/Lead Optimization phase varied from inability to optimize both activity and metabolic stability into the same molecule to the need to develop a more robust animal model, which was out of the scope and budget of the BPN resources. To date, two projects have graduated from the program successfully. The Familial Dysautonomia project at Massachusetts General Hospital was licensed and internalized in the preclinical phase [7, 8]. The second project, from Tetra Discovery Partners, successfully completed all milestones including successful completion of the phase I Single Ascending Dose study as part of the BPN program and is advancing further via additional NIH and external funding resources [9–12]. The remaining 3 active programs from the original 15 are advancing through preclinical activities in preparation for filing Investigational New Drug applications to enable phase I trial in 2017. Consistent with the goal to move from NIH-funded, to industry-funded development, the Eolas project started with Scripps Florida, then transferred from the academic institution to Eolas, a company founded by the principal investigators, and successfully entered a worldwide license and partnership agreement with AstraZeneca [13, 14]. Additionally, the Columbia Dry Age-Related Macular Degeneration project became the most recent project to announce a licensing agreement as it prepares to enter the clinic [15].

In conclusion, during the first 5 years, the BPN exceeded the goals for the program, and overall advancement of projects is in-line with industry average attrition [4]. These milestone-driven cooperative agreements allowed for timely decisions while focusing valuable NIH resources and grant funding on the most promising projects. Importantly, projects starting in the academic setting were well represented at every stage of the BPN program. From the original 15 projects, 1 project completed its phase I trial and progressed to additional clinical trials, and 4 licensing agreements were announced to date. Additional projects are poised to enter the clinic in the near future. In light of the BPN pilot program’s success, it was decided to continue the project for an additional 5 years. Several Funding Opportunity Announcements were generated by the National Institute of Neurological Disorders and Stroke in conjunction with other NIH Blueprint for Neuroscience Research ICs with the goal of helping to ensure project preparedness in the early stages of the neuroscience translational research and to ensure the resources to discover, develop, and advance promising small molecules from academic laboratories and small businesses to phase I clinical trials. (See IGNITE and BPN Funding Opportunity Announcements http://www.ninds.nih.gov/funding/areas/translational_research/funding_programs_researchers.htm). This suite of grant mechanisms, supported by experienced program managers at NIH and contract resources, are available to the neuroscientific community. Investigators are encouraged to make use of these resources to translate their discoveries to therapies that will advance health.

## Electronic supplementary material

Below is the link to the electronic supplementary material.

Required Author Forms Disclosure forms provided by the authors are available with the online version of this article. (PDF 1192 kb)

## References

[1] Abbott A (2011). Novartis to shut brain research facility. Nature.

[2] Stovall, S. R&D cuts curb brain-drug pipeline: development of new medicines for brain disorders could be threatened as major drug makers scale back research. Wall Street Journal; March 27, 2011. Available at: http://www.wsj.com/articles/SB10001424052748704474804576222463927753954 Accessed February 8, 2017.

[3] Heemskerk J, Farkas R, Kaufmann P (2012). Neuroscience networking: linking discovery to drugs. Neuropsychopharmacology.

[4] Brown D, Superti-Furga G (2003). Rediscovering the sweet spot in drug discovery. Drug Discov Today.

[5] Funding was continued via a R01 grant and resulted in funding from outside sources and formation of a new company. Available at: http://www.oricularx.com. Accessed August 7, 2016.

[6] Reset Therapeutics, Inc. establishes research collaboration with Alkermes for discovery and development of novel orexin modulators. Available at: http://www.businesswire.com/news/home/20160317005378/en/. Accessed March 17, 2016.

[7] Massachusetts General Hospital. Optimization of compounds to improve mRNA splicing in familial dysautonomia (PI: Susan Slaugenhaupt). Available at: https://projectreporter.nih.gov/project_info_description.cfm?aid=8918034&icde=30204642. Accessed March 28, 2017.

[8] PTC therapeutics and massachusetts general hospital collaborate on rare disease research. Available at: http://ir.ptcbio.com/releasedetail.cfm?releaseid=946252. Accessed December 9, 2015.

[9] Tetra Discovery Partners. PDE4D allosteric modulators for treating cognitive impairment (PI: Mark Gurney). Available at: https://projectreporter.nih.gov/project_info_description.cfm?aid=9068253&icde=30204682. Accessed March 28, 2017.

[10] Tetra Discovery advances Alzheimer's treatment candidate BPN14770 to phase i multiple ascending dose trial. Available at: http://www.prnewswire.com/news-releases/tetra-discovery-advances-alzheimers-treatment-candidate-bpn14770-to-phase-i-multiple-ascending-dose-trial-300244787.html. Accessed April 1, 2016.

[11] Tetra Discovery Partners raises $10 million through series A financing and two National Institutes of Health grants. Available at: http://www.prnewswire.com/news-releases/tetra-discovery-partners-raises-10-million-through-series-a-financing-and-two-national-institutes-of-health-grants-300369553.html?tc=eml_cleartime. Accessed November 29, 2016.

[12] Tetra Discovery Partners announces positive results from phase 1 studies of cognition drug candidate, BPN14770. Available at: http://www.prnewswire.com/news-releases/tetra-discovery-partners-announces-positive-results-from-phase-1-studies-of-cognition-drug-candidate-bpn14770-300379985.html. Accessed December 19, 2016.

[13] Eolas Therapeutics. Orexin receptor antagonists for drug addiction and panic disorder (PI: Paul Kenny). Available at: https://projectreporter.nih.gov/project_info_description.cfm?aid=8856678&icde=30204721. Accessed March 28, 2017.

[14] Eolas Therapeutics and AstraZeneca partner to develop orexin-1 receptor antagonist for multiple indications. Available at: http://www.prnewswire.com/news-releases/eolas-therapeutics-and-astrazeneca-partner-to-develop-orexin-1-receptor-antagonist-for-multiple-indications-300106505.html. Accessed June 30, 2015.

[15] Lin Bioscience licenses first-in-class therapeutic program to treat dry aged-related macular degeneration from Columbia University in collaboration with NIH. Available at: http://www.prnewswire.com/news-releases/lin-bioscience-licenses-first-in-class-therapeutic-program-to-treat-dry-aged-related-macular-degeneration-from-columbia-university-in-collaboration-with-nih-300386708.html#continue-jump. Accessed January 6, 2017.

